# Patterns of occupational commitment among nurses: a latent profile analysis

**DOI:** 10.3389/fpsyg.2024.1331425

**Published:** 2024-05-30

**Authors:** Zihan Lin, Wenbin Wu, Huifang Zhang, Zhiqiang He, Mengyu Han, Jin Li

**Affiliations:** ^1^Department of Nursing, Health Science Center, Xi’an Jiaotong University, Xi’an, China; ^2^The First Affiliated Hospital of Xi’an Jiaotong University, Xi’an, China

**Keywords:** nurses, occupational commitment, latent profile analysis, psychological empowerment, job crafting, job crafting workforce

## Abstract

**Background:**

Occupational commitment (OC) is a multidimensional construct that predicts turnover intentions. The interindividual variability of nurses’ OC merits further exploration. Therefore, this study aims to examine patterns of OC and its relationship with psychological empowerment and job crafting in nurses.

**Methods:**

A sample of 1,061 nurses was recruited from February 2022 to April 2022 by using a stratified four-stage cluster sampling procedure. A self-report survey included the Psychological Empowerment Scale, Job Crafting Scale, and Occupational Commitment Scale. Latent profile analysis (LPA) was used to examine the patterns of OC. Associations of the latent class membership with individual characteristics, psychological empowerment and job crafting were examined using multinomial logistic regression.

**Results:**

Three patterns of OC were identified: (1) “Low OC group” (*n* = 224, 21.1%); (2) “Moderate OC group” (*n* = 665, 62.7%); (3) “High OC group” (*n* = 172, 16.2%). Nurses with higher education, fewer years of service, working in medicine, lower psychological empowerment and lower job crafting had a higher likelihood of belonging to Class 1 (Low OC group). In contrast, nurses working in emergency and with higher psychological empowerment and job crafting were more likely to belong to Class 3 (High OC group).

**Conclusion:**

The findings revealed the heterogeneity of occupational commitment among nurses in China and could guide the identification and early intervention of nurses with low level of occupational commitment.

## 1 Introduction

Mainland China is struggling with a severe shortage of nurses similar to the rest of the world (Wang et al., 2012). The current global pandemic has greatly exacerbated these problems, which are evident in the disturbing rates of turnover and turnover intention among nurses (Falatah, 2021). A recent study that surveyed 63,947 nurses found that 63.4% of nurses had a high turnover intention in China (Liu et al., 2021). In an era of unprecedented turnover of nurses of all types, retaining nurses is especially important. Many studies have highlighted the importance of understanding the mechanisms of nurse turnover intention (Sasso et al., 2019; Cao et al., 2021; Kelly et al., 2021), and perhaps more importantly, what factors are positively correlated with their occupational commitment (OC).

OC is more than a commitment to the particular organization and implies individuals’ view toward their career and the motivation that they have to stay in their jobs (Gallagher and Parks, 2001). It is very important to understand the OC of nurses, because it can reflect nurses’ attitude comprehensively and permanently from the occupational level, and has a good predictive effect on individual turnover intention (Jourdain and Chênevert, 2010; Wang et al., 2012). Although aggregate scores indicate overall OC, different types of OC are equally important because these individuals have different risk factors and consequences. Whether there are different OC clusters among nurses and how to identify these clusters are worth exploring.

## 2 Literature

Research on workplace commitment dates back to the 1960s and has focused primarily on the concept of organizational commitment (Cohen, 2011). Organizational commitment refers to a psychological state that binds the individual to the organization (Martins et al., 2023). With the current workforce becoming increasingly complex and flexible, people can no longer assume that organizational commitment is the only or primary commitment in the workplace (Cohen, 2011). For most people, occupation is a necessary part of their life and livelihoods. Organizational restructurings, also involving health care and nursing, and the attendant insecurity has shifted the interest of employees and researchers from organizational commitment to OC (Numminen et al., 2016). OC refers to the individuals’ perception of their profession, their willingness to pursue their professional values and goals and their motivation to remain in their occupation (Siraneh et al., 2018). It is not just a commitment to a job or an organization, but a psychological connection between a person and his or her occupation based on an affective reaction to that occupation. A person with a strong OC actively identifies with his or her occupation, while identifying with the larger group by sharing its values and beliefs (Ng, 2015). According to the study by Blau (2003) OC consists of four dimensions: (1) affective commitment, (2) normative commitment, (3) accumulated costs commitment and (4) limited alternatives commitment.

To date, most research studying the four components of OC has used between-persons, variable-centered analysis. Variable-centered analysis is appropriate when the goal is to understand how the four commitment components vary in a population or when the objective is to capture relationships among a limited set of variables within a group of individuals (Meyer et al., 2013). However, an emerging trend is to consider how these four components are experienced by the individual as a whole, and whether the behavioral impact of any one component is dependent on the relative strength or combination of the other components. Though variable-centered analysis has served the commitment literature well by elucidating inter-individual differences, person-centered analysis (e.g., Latent profile analysis) offer the opportunities to capture the nuance and complexity of inter-individual variation in variable systems (Meyer et al., 2013).

Rather than focusing on components of individuals, person-centered analysis identifies and compares subgroups of individuals who share similar patterns on the four components (Meyer and Herscovitch, 2001). When similar individuals are grouped together, researchers can examine in a theoretically predictable manner whether different profile groups differ on one or more criteria. Latent profile analysis (LPA) is rather novel in the OC research among nurses, but it has been shown to be valid and meaningful for exploring the subtypes of organization commitment (Gellatly et al., 2014) and turnover intention (Chen et al., 2015) in nurses. Therefore, LPA can be used to identify the patterns of OC in nurses.

According to the Contextual Work-Life Experiences Model (CWLEXP) proposed by Dilmi et al. in 2018, nurses’ immediate work context (e.g., quality of work itself and resources nature of job) and individual characteristics play a key role in OC (Aluwihare-Samaranayake et al., 2018). Therefore, the immediate work context and individual characteristics associated with the OC of nurses might be essential for identifying the patterns. At present, many studies have investigated several demographic variables that influence nurses’ OC, including gender, age, educational level and marital status (Azim and Islam, 2018; Onyishi et al., 2019).

Regarding the psychological and behavioral factors that reflect work context, psychological empowerment and job crafting were also a focus of this study. Psychological empowerment is recognized as employees’ psychological perception or attitude toward their work and their organizational roles (Spreitzer, 1995). It integrates self-determination and competence from intrinsic motivation theory and meaning and impact from job characteristics theory (Wang and Zhang, 2017). The development of psychological empowerment enhances organizational commitment and predicates turnover intention (Shapira-Lishchinsky and Benoliel, 2019; Albasal et al., 2022). Job crafting is defined as the physical and cognitive changes in individuals within the context of the task or relational boundaries of their work (Moreira et al., 2022). This is proactive behavior of employee designed to improve the work environment and optimize the fit between individuals’ abilities/preferences and the job (Tims and Bakker, 2010; Bakker, 2018). Therefore, exploring the features of psychological empowerment and job crafting in relation to different subtypes of nurses’ OC can identify the target populations for precise intervention.

The aim of this study is to investigate the level of OC among Chinese nurses, identify the patterns of OC and explore the characteristics of different OC clusters in terms of demographic factors, psychological empowerment and job crafting. The main hypotheses were as follows: (a) different subtypes of OC exist among these nurses; and (b) the demographic characteristics, level of psychological empowerment and job crafting are different among the subgroups.

## 3 Methods

### 3.1 Study design

A cross-sectional study was conducted using a stratified four-stage cluster sampling procedure. In Stage 1, according to the geographical location, Shaanxi was divided into Northern Shaanxi (including three cities), Guanzhong (including five cities) and Southern Shaanxi (including two cities). In Stage 2, selected cities in proportion from the above three regions randomly, and finally selected a total of four cities (one each in Southern Shaanxi and Northern Shaanxi, two in Guanzhong). In Stage 3, one Class III Grade A comprehensive hospital was randomly selected from each city. In Stage 4, nurses were randomly selected proportionally from the departments of medicine, surgery, obstetrics/gynecology, pediatrics, emergency, oncology, and ICU/CCU in each hospital. The study complied with the STROBE Checklist of items.

### 3.2 Procedure and participants

Survey data were collected from four Class III Grade A comprehensive hospitals located in four cities in Shaanxi province from February 2022 to April 2022. To ensure comparability of survey data, the four hospitals were similar in terms size, grade and health care services provided. The inclusion criteria for the participants were full-time nurses who have passed the probationary period. First, the researchers contacted the nursing directors at each hospital, and then, the nursing directors distributed the online link^1^ of the questionnaire to the nurses who met the inclusion criteria.

Based on the descriptive study sample size, a sample size of five to ten times the number of items was generally required. The total number of items in the study was 68 (11 on demographic characteristics, 12 on PES, 21 on JCS, 24 on OCS). And assuming an invalid response rate of 30%, the sample size required for study was 442–884. Initially, 1,146 participants received and completed the questionnaire, after excluding participants with short response time and regular responses, 1,061 valid responses were collected (valid rate was 92.6%). Among 1,061 participants, 433 were working in Xi’an, 175 in Weinan, 241 in Hanzhong and 212 in Yan’an.

### 3.3 Instruments

#### 3.3.1 Demographic characteristics

A demographic information form was developed drawing from the literature on factors associated with nurses’ OC. It included items about age, sex, marital status, number of children, level of education, monthly income, work unit, length of service, professional title, type of employment and monthly number of night shifts.

#### 3.3.2 Occupational commitment scale (OCS)

OC was assessed using the Chinese version of the Occupational Commitment Scale (OCS), which was developed by Blau (2003). In the previous study, all items were translated into Chinese and applied to the nurses by Pei et al. (2007). The Chinese version of the scale had 24 items and contained five dimensions: affective commitment (items 1 ~ 6), normative commitment (items 7 ~ 11), economic cost commitment (items 12 ~ 15), emotional cost commitment (items 16 ~ 20), and limited alternatives commitment (items 21 ~ 24). Each item was scored on a five-point Likert scale from 1 (strongly disagree) to 5 (strongly agree), and the last dimension was scored in reverse. In this study, the scale showed a good reliability (Cronbach’s alpha = 0.940).

#### 3.3.3 Psychological empowerment scale (PES)

The Psychological Empowerment Scale (PES), developed by Spreitzer (1995), was used to assess psychological empowerment. This 12-item scale consists of 4 sub-scale: meaning (items 1, 5, 9), self-determination (items 2, 6, 10), competence (items 3, 7, 11), and impact (item 4, 8, 12). Each item is rated from 1 to 5, and the scores of each subscale range from 3 to 15. High scores indicate better psychological empowerment. The Chinese version of the PES showed good reliability and validity (Li et al., 2006). Cronbach’s alpha was 0.922 in this sample.

#### 3.3.4 Job crafting scale (JCS)

The scale for job crafting developed by Tims et al. (2012) and translated into Chinese by Liao (2013) was used to assess nurses’ job crafting. This measure is a 21-item self-report scale classified into four dimensions: increasing structural job resources (items 1 ~ 5), decreasing hindering job demands (items 6 ~ 11), increasing social job resources (items 12 ~ 16), and increasing challenging job demands (17 ~ 21). Each item was rated on 5-point Likert scale from 1 (never) to 5 (always), with higher scores indicating higher job crafting. The JCS demonstrated satisfactory reliability. Cronbach’s alpha was 0.943 in this study.

### 3.4 Statistical analysis

SPSS version 26.0 and Mplus version 8.3 were used for statistical analysis. Data for the five dimensions of OCS were fed into the LPA, initially as one class and then progressively adding other classes until a unique solution could not be determined using the maximum likelihood approach. The fit indices were tested. The Akaike information criterion (AIC), Bayesian information criterion (BIC) and adjusted BIC (aBIC) were used to indicate the best fit with the lowest value (Peugh and Fan, 2013). The Lo–Mendell–Rubin (LMR) and bootstrapped likelihood ratio test (BLRT) were applied to assess the *p*-values in the comparisons between models. A significant *p* value indicates that the k-class model fits the data better than the k-1-class model (Stanley et al., 2017). The entropy values were used to evaluate the separability of each LPA solution, and values closer to 1 indicate a better separation of classes (Asparouhov and Muthen, 2014). In addition, the average posterior probability should greater than 0.70 indicates that respondents belong to the assigned profile and no other profiles (Stanley et al., 2017).

Subsequently, chi-square test or Fisher’s exact test and analysis of variance were used to examine whether demographic characteristics distinguish the classes. Variables with *p* < 0.1 in chi-square test were included in multinomial logistic regression. Multinomial logistic regression was performed to evaluate the association of demographic characteristics, psychological empowerment and job crafting with class membership. All statistical tests were two-tailed, and a *p* value of <0.05 was considered statistically significant in multinomial logistic regression.

### 3.5 Ethical considerations

Ethics approval for the study was obtained from the Ethics Committee of Xi’an Jiaotong University Health Science Center (No. 2022–1175). During the investigation, the principle of voluntary participation, confidentiality and non-harm shall be strictly observed.

## 4 Results

### 4.1 Participants’ demographic characteristics

The demographic characteristics of participants included in analyses are shown in Table 1. Most participants were female (98.2%), and the mean age was 33.42 (SD = 6.01). The majority was married (78.1%) with bachelor or above education (79.2%) and a monthly income of ≤5,999 RMB won (73.6%). Nurses working at surgery accounted for 35.8% of the total participants, and nurses at medicine accounted for 31.1%. The average length of service was 11.08 years (range: 0.5 ~ 38).

**Table 1:** Characteristics of participants (*n* = 1,061).

Characteristics	*n*	%	Characteristics	*n*	%
Age			Work unit		
≤25	79	7.4	Medicine	330	31.1
26 ~ 30	291	27.4	Surgery	380	35.8
31 ~ 40	582	54.9	Obstetrics/gynecology	75	7.1
≥41	109	10.3	Pediatrics	133	12.5
Sex			Emergency	59	5.6
Female	1,042	98.2	Oncology	42	4.0
Male	19	1.8	ICU/CCU	42	4.0
Marital status			Length of service		
Married	829	78.1	≤5 years	205	19.3
Others	232	21.9	6 ~ 10 years	351	33.1
Number of children			11 ~ 20 years	424	40.0
0	298	28.1	≥21 years	81	7.6
1	508	47.9	Professional title		
≥2	255	24.0	Nurse	193	18.2
Level of education			Nurse practitioner	471	44.4
College or below	221	20.8	Nurse-in-charge or above	397	37.4
Bachelor or above	840	79.2	Monthly number of night shifts		
Monthly income			0	303	28.6
≤5999RMB	781	73.6	1 ~ 4	159	15.0
≥6000RMB	280	26.4	5 ~ 8	302	28.5
Type of employment			≥9	297	28.0
Contract basis	892	84.1			
Official	169	15.9			

### 4.2 Latent profile analysis

Table 2 shown the latent class solution of OC in nurses. Based on the model fit statistics, the optimal number of latent classes was determined. Although the five-class model appeared to be the optimal model, the sample size of one class was small (*n* = 23). The three-class model had relatively low AIC, BIC, and aBIC values; relatively high entropy; and significant LMR and BLRT values. In addition, the three-class model provided good class membership classification and the most interpretable information from a clinical perspective. The results of item-response probability in the three-class model showed that the average probability of the nurses in each potential category was between 95.8 and 97.0%, which support the reliability of the model.

**Table 2:** Fit indices for class models 1 through 5 (*n* = 1,061).

Model	AIC	BIC	aBIC	Entropy	LMR-LRT *p* value	BLRT *p* value
1-Class	28690.338	28740.007	28708.246	-	-	-
2-Class	27337.341	27416.812	27365.993	0.839	<0.001	<0.001
3-Class	26115.171	26224.444	26154.568	0.938	<0.001	<0.001
4-Class	25684.072	25823.147	25734.214	0.942	0.153	0.158
5-Class	25240.234	25409.111	25301.121	0.957	0.045	0.048

-, not applicable.

AIC, Akaike information criterion; BIC, Bayesian information criterion; aBIC, adjusted BIC; LMR-LRT, Lo–Mendell–Rubin-likelihood ratio test; BLRT, bootstrapped likelihood ratio test.

**Figure 1:**
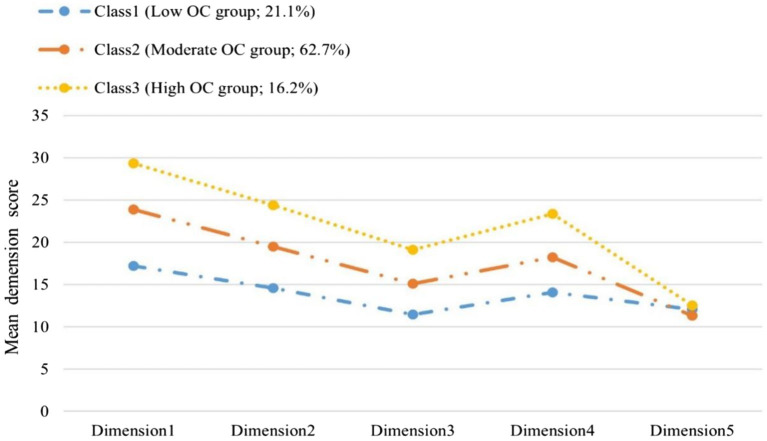
Depiction of three latent classes defined by patterns of means for OCS.

Three profiles of each dimension are depicted in Figure 1. Class 1 (*n* = 224, 21.1%) was characterized by the lowest and lower scores for the five dimensions. The values of the first four dimensions were all lower than the mean of the entire sample for each of the domains considered. Overall, members of this profile had perceptions about their low OC. We called this profile “Low OC group.” Class 2 accounted for 62.7% (*n* = 665) of the sample. It was characterized by scores close to the average for all five domains. Members of this profile had perceptions about their moderate OC. We labeled this class “Moderate OC group.” Class 3 comprised the least people (*n* = 172, 16.2%) had the highest probabilities of maintaining OC, and was classified into the “High OC group.”

### 4.3 Difference in characteristics among the latent classes

There were significant differences in level of education and work unit among three latent profiles (both *p* < 0.05, Table 3). In addition, OC, psychological empowerment, job crafting and their dimensional scores differed significantly among the three potential profiles (both *p* < 0.05, Table 4).

**Table 3:** Comparison of OC of the classes by demographic variables.

Variables	Class 1 (*n* = 224)	Class 2 (*n* = 665)	Class 3 (*n* = 172)	*p*
Age				0.12
≤25	13 (5.80)	53 (7.97)	13 (7.56)	
26 ~ 30	66 (29.46)	183 (27.52)	42 (24.42)	
31 ~ 40	133 (59.38)	351 (52.78)	98 (56.98)	
≥41	1 (5.36)	78 (11.73)	19 (11.05)	
Sex				0.30
Female	218 (97.32)	653 (98.20)	171 (99.42)	
Male	6 (2.68)	12 (1.80)	1 (0.58)	
Marital status				0.35
Married	169 (75.45)	529 (79.55)	131 (76.16)	
Others	55 (24.55)	136 (20.45)	41 (23.84)	
Number of children				0.25
0	74 (33.04)	179 (26.92)	45 (26.16)	
1	94 (41.96)	332 (49.92)	82 (47.67)	
≥2	56 (25.00)	154 (23.16)	45 (26.16)	
Level of education				0.04
College or below	34 (15.18)	144 (21.65)	43 (25.00)	
Bachelor or above	190 (84.82)	521 (78.35)	129 (75.00)	
Monthly income				0.30
≤5999RMB	174 (77.68)	483 (72.63)	124 (72.09)	
≥6000RMB	50 (22.32)	182 (27.37)	48 (27.91)	
Work unit				0.02
Medicine	86 (38.39)	202 (30.38)	42 (24.42)	
Surgery	67 (29.91)	238 (35.79)	75 (43.60)	
Obstetrics/gynecology	17 (7.59)	43 (6.47)	15 (8.72)	
Pediatrics	33 (14.73)	78 (11.73)	22 (12.79)	
Emergency	11 (4.91)	38 (5.71)	10 (5.81)	
Oncology	3 (1.34)	36 (5.41)	3 (1.74)	
ICU/CCU	7 (3.13)	30 (4.51)	5 (2.91)	
Length of service				0.06
≤5 years	39 (17.41)	130 (19.55)	36 (20.93)	
6 ~ 10 years	79 (35.27)	214 (32.18)	58 (33.72)	
11 ~ 20 years	100 (44.64)	261 (39.25)	63 (36.63)	
≥21 years	6 (2.68)	60 (9.02)	15 (8.72)	
Professional title				0.41
Nurse	46 (20.53)	110 (16.54)	37 (21.51)	
Nurse practitioner	93 (41.52)	301 (45.26)	77 (44.77)	
Nurse-in-charge or above	85 (37.95)	254 (38.20)	58 (33.72)	
Type of employment				0.44
Contract basis	191 (85.27)	552 (83.01)	149 (86.63)	
Official	33 (14.73)	113 (16.99)	23 (13.37)	
Monthly number of night shifts				0.13
0	56 (25.00)	197 (29.62)	50 (29.07)	
1 ~ 4	28 (12.50)	102 (15.34)	29 (16.86)	
5 ~ 8	65 (29.02)	198 (29.77)	39 (22.67)	
≥9	75 (33.48)	168 (25.26)	54 (31.40)	

**Table 4:** Comparison of the different classes by OC, psychological empowerment and job crafting.

Variables	Class 1 (*n* = 224) Mean (SD)	Class 2 (*n* = 665) Mean (SD)	Class 3 (*n* = 172) Mean (SD)	*p*
OC				
Affective commitment	17.15 (3.61)	23.88 (2.32)	29.31 (1.75)	<0.01
Normative commitment	14.52 (2.36)	19.52 (1.67)	24.39 (1.01)	<0.01
Economic cost commitment	11.49 (2.43)	15.06 (1.53)	19.12 (1.28)	<0.01
Emotional cost commitment	14.05 (3.11)	18.19 (2.99)	23.41 (2.45)	<0.01
Limited alternatives commitment	12.05 (2.61)	11.30 (2.80)	12.56 (5.09)	<0.01
Total	69.25 (7.89)	87.95 (5.37)	108.79 (6.24)	<0.01
Psychological empowerment				
Meaning	9.97 (2.31)	12.12 (1.69)	13.88 (2.01)	<0.01
Self-Determination	9.16 (2.50)	10.99 (2.10)	12.47 (2.81)	<0.01
Competence	11.18 (2.59)	12.56 (1.58)	13.98 (1.88)	<0.01
Impact	7.88 (2.23)	9.60 (2.38)	10.63 (3.46)	<0.01
Total	38.18 (7.86)	45.28 (6.11)	50.95 (8.34)	<0.01
Job crafting				
Increasing structural job resources	18.10 (3.48)	20.67 (2.41)	23.60 (2.18)	<0.01
Decreasing hindering job demands	19.47 (3.49)	21.92 (3.46)	25.34 (4.83)	<0.01
Increasing social job resources	15.73 (2.92)	18.61 (2.88)	22.37 (3.28)	<0.01
Increasing challenging job demands	15.19 (3.25)	18.37 (2.91)	22.39 (3.13)	<0.01
Total	68.49 (10.29)	79.58 (8.86)	93.70 (11.04)	<0.01

### 4.4 Multinomial logistic regression analysis

**Figure 2:**
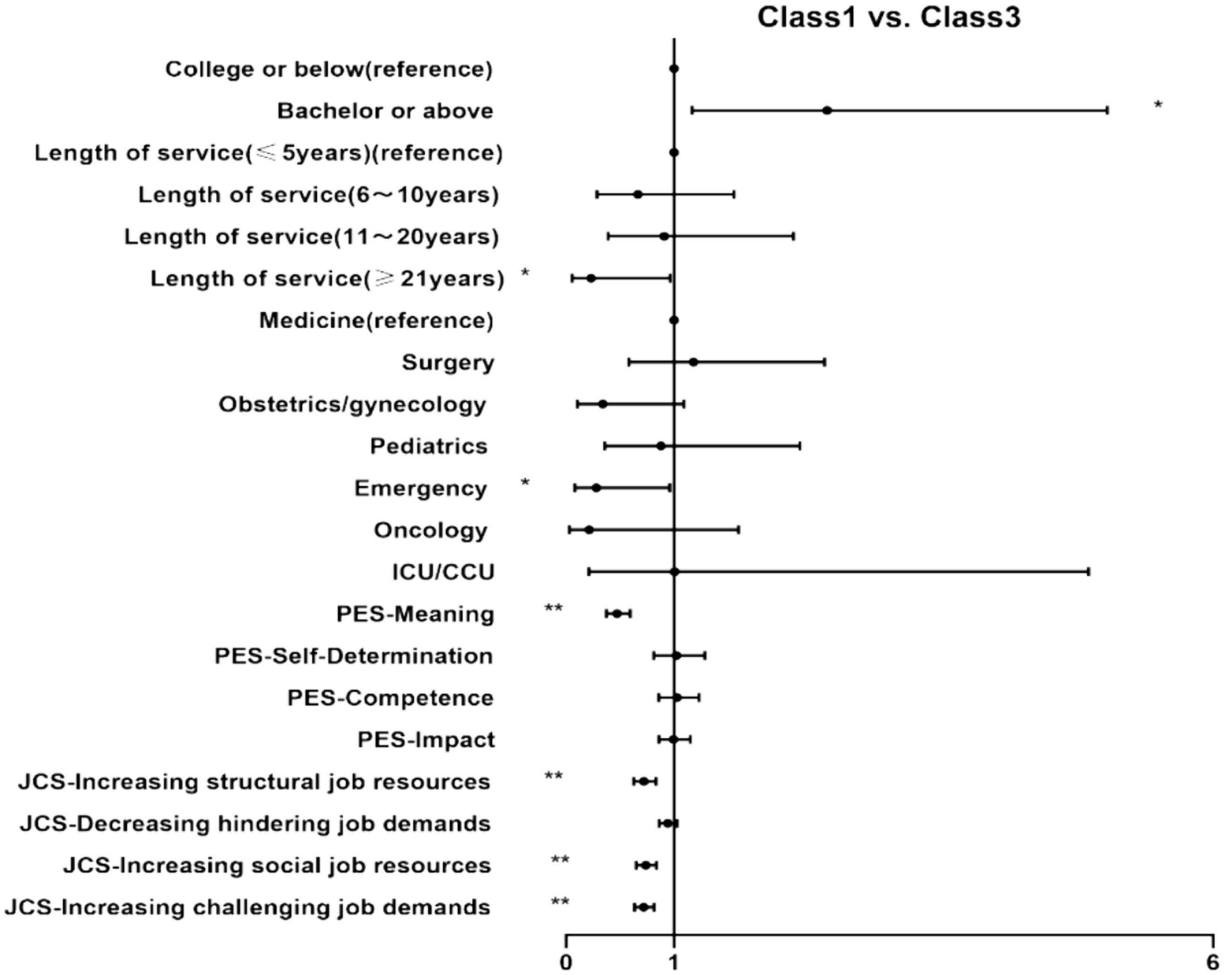
MLRA showing the association of (1) demographics, (2) psychological empowerment and (3) job crafting with Class 1, compared to Class3. *p < 0.05 **p < 0.01. MLRA, multinomial logistic regressions. Class 1: Low OC group; Class 3: High OC group.

**Figure 3:**
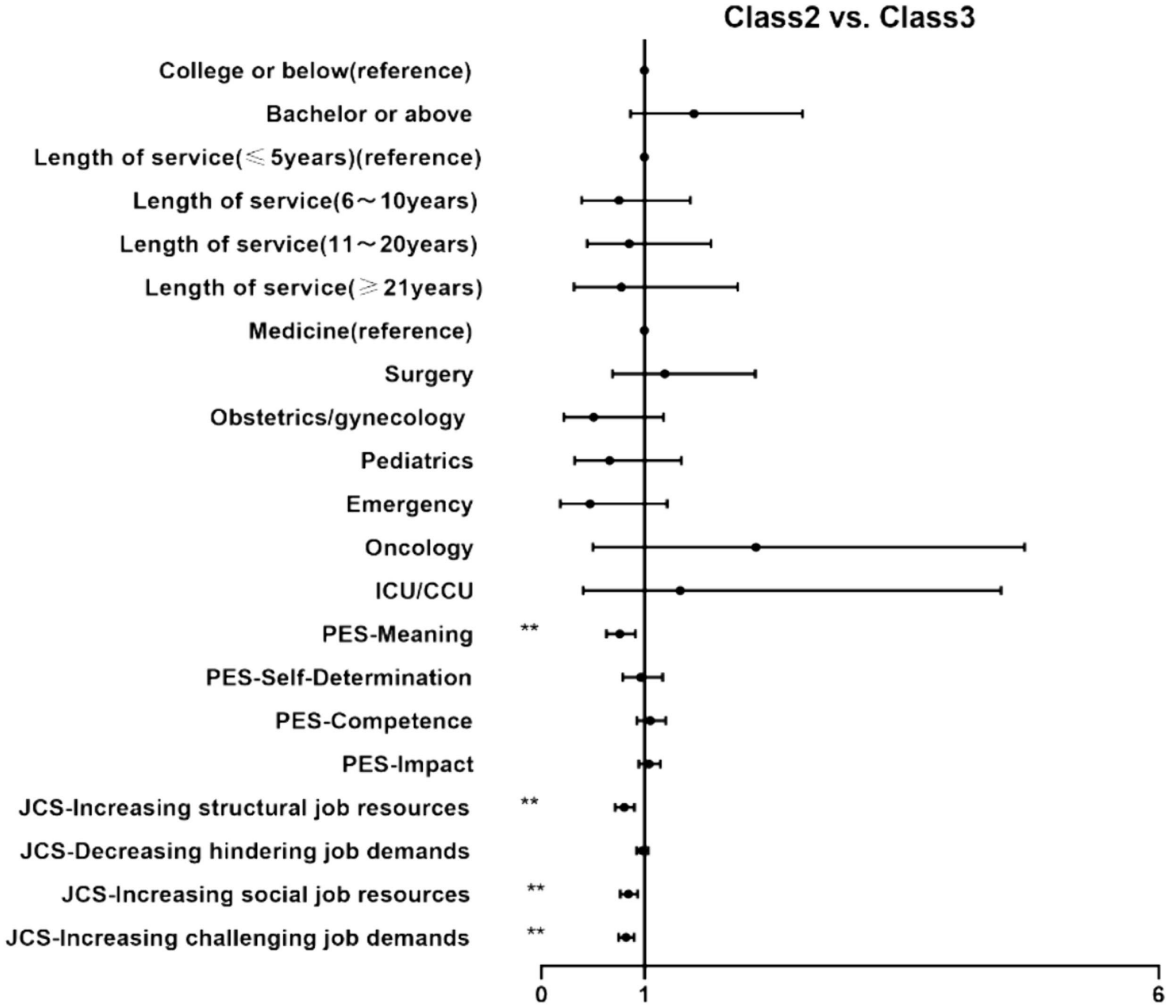
MLRA showing the association of (1) demographics, (2) psychological empowerment and (3) job crafting with Class 2, compared to Class3. *p < 0.05 **p < 0.01. MLRA, multinomial logistic regressions. Class 2: Moderate OC group; Class 3: High OC group.

**Figure 4:**
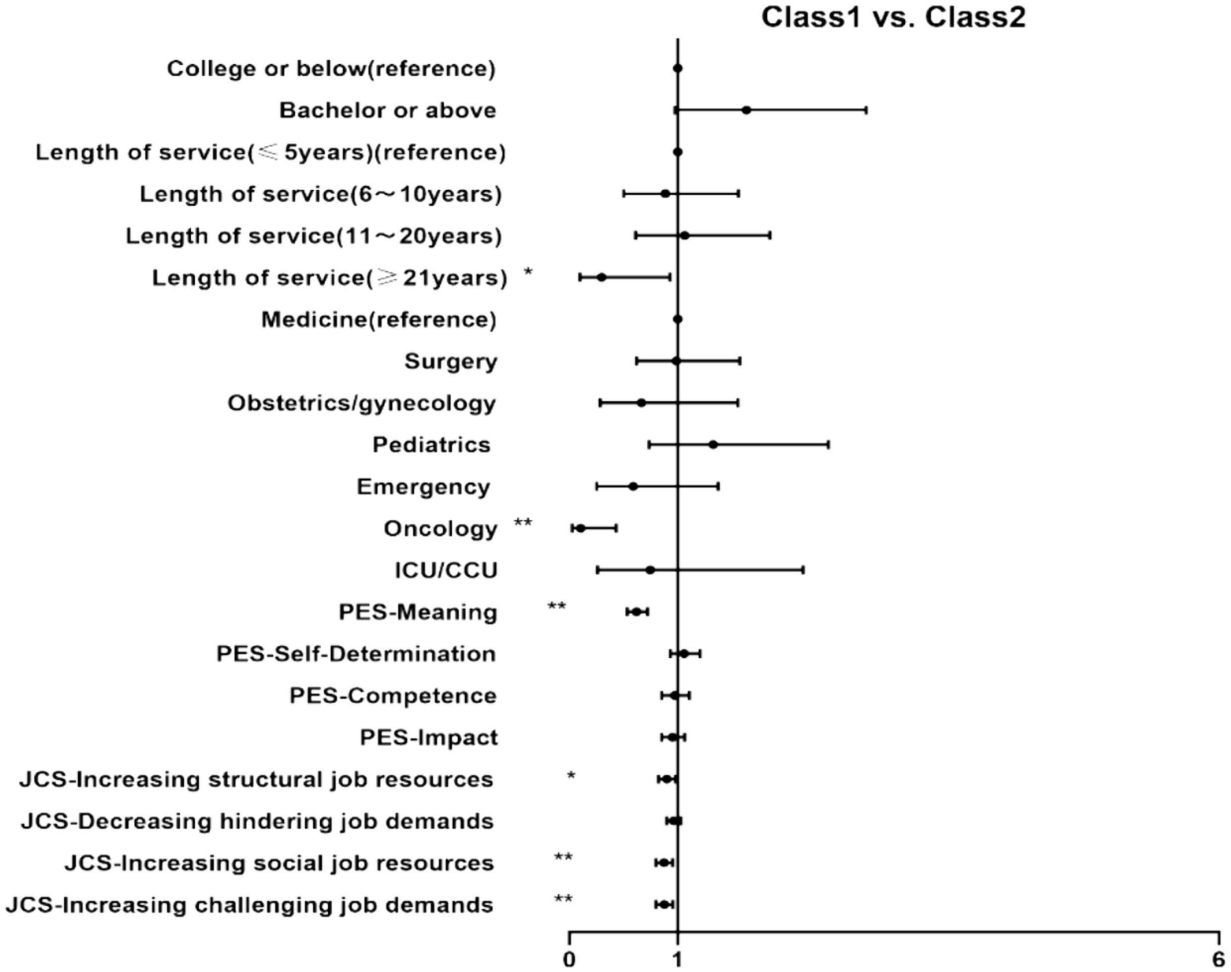
MLRA showing the association of (1) demographics, (2) psychological empowerment and (3) job crafting with Class 1, compared to Class3. *p < 0.05 **p < 0.01. MLRA, multinomial logistic regressions. Class 1: Low OC group; Class 2: Moderate OC group.

We conducted a multinomial logistic regression analysis, classes were compared with each other, and the results are shown in Figures 2–4.

First, Class 3 (high OC group, as a reference) was compared with the other classes (Figures 2, 3). High meaning (OR = 0.470–0.760), increasing structural job resources (OR = 0.721–0.803), increasing social job resources (OR = 0.737–0.845) and increasing challenging job demands (OR = 0.717–0.821) levels had significant negative effects on the odds of being in Class 1 and Class 2 than in Class 3. People with bachelor or above increased probability of belonging to Class 1 (OR = 2.420, 95% CI: 1.167–5.017), while more years of service decreased the probability (OR = 0.055, 95% CI: 0.230–0.963). In addition, compared with medicine, nurses work at emergency were less likely to be categorized into Class 1 than Class 3 (OR = 0.277, 95% CI: 0.080–0.959).

Second, Class 2 (moderate OC group, as a reference) was compared with Class 1 (Figure 4). The probability of belonging to Class 1 was lower with higher meaning (OR = 0.619, 95% CI: 0.532–0.721), increasing structural job resources (OR = 0.898, 95% CI: 0.822–0.980), increasing social job resources (OR = 0.872, 95% CI: 0.799–0.952) and increasing challenging job demands (OR = 0.874, 95% CI: 0.799–0.955) levels. Compared with ≤5 years of service (reference group), more years of service (≥21 years) were less likely to be grouped into Class 1 (OR = 0.297, 95% CI: 0.095–0.928). And, compared with medicine, nurses work at oncology had a lower chance of belonging to Class 1 than Class 2 (OR = 0.102, 95% CI: 0.024–0.430).

## 5 Discussion

Interest in commitment profiles has increased dramatically. The present study examined profiles of OC and the association with psychological empowerment and job crafting in a multicenter study involving 1,061 nurses.

### 5.1 OC of nurses

LPA identified three different classes based on the model accuracy indices and clinical practical significance. Approximately 21.1% of the participants were classified in “Low OC group,” 62.7% were in “Moderate OC group” and 16.2% in “High OC group.” Substantial differences in five dimensions were found in the three classes. Our findings support the hypothesis that there are different subtypes of OC among nurses. Notably the characteristics of the members in the three classes reported highest scores in affective commitment and relatively low scores in limited alternatives commitment, which was consist with the previous study (Somers et al., 2019). That indicated that most nurses identified with nursing at the emotional level, which may be related to the popularity of higher nursing education in China and the focus on education for professional identity by nursing colleges and hospitals. The low level of limited alternatives commitment showed that nurses were dissatisfied with their current treatment and status. With the improvement of their comprehensive quality, they will consider leaving the profession and choose a more ideal career in the face of heavy tasks and fierce competition in work environment.

### 5.2 Demographic characteristics of the different classes

Given that OC are highly heterogeneous, demographic characteristics have certain specific in predicting subtypes. For instance, presence of the Class 1 (Low OC group) was relatively likely to be nurses with lower years of service and higher education level compared with the other classes. Consistent with previous studies (Sonmez and Benligiray, 2013; Onyishi et al., 2019), low education statue and tenure have significant positive correction with OC. Better educational status may provide individuals with more satisfying job opportunities and a great likelihood of obtaining the career they want and choose. While, as the years of service increase, so does the nursing experience. Nogueras revealed that more work experience leads to stronger OC (Nogueras, 2006). Moreover, compared with medicine, nurses working in oncology and emergency were more likely to belong to Class 2 and Class 3, respectively. That indicated that emergency nurses recognized their profession and had a strong motivation to stay in this profession, while medicine nurses were the opposite. This is very interesting and could be explored further in future studies.

### 5.3 Psychological and behavioral factors of the different classes

Regarding the differences in psychological empowerment among groups, Class 3 showed the highest level of psychological empowerment, which confirmed its association with OC. There were no significant differences in four dimensions among the three classes, except for meaning. That indicated the perception of meaning of the tasks is the most important for nurses’ commitment, which is similar to previous studies (Joo and Shim, 2010; Sumi, 2011). Put differently, nurses will have higher OC when the work goal or purpose is met with individual value. However, nurses with higher degree of self-determination, competence, and impact will have enough confidence that they do not guarantee their commitment toward their profession and may leave the profession if the need arises. The findings were consistent with other previous studies that not all psychological empowerment dimensions have positive correlation with work outcomes. For example, Sumi found that only meaning and self-determination dimension have positive relationship with organizational commitment (Sumi, 2011), while Li proved competence was not related to job satisfaction (Li et al., 2013). On the other hand, influence of age (low age and high age) in relationship between psychological empowerment and work engagement has been conducted (Juyumaya, 2022). Their findings pointed out that, the relationship between psychological empowerment and work engagement is positive for young employees, but not for older employees.

Given the differences in the OC and job crafting among the three classes, the results show significant differences in the domains of increasing structural job resources, increasing social job resources and increasing challenging job demands. In the process of seeking resources, nurses will improve the fit between degree of job and competencies, work styles and hobbies (Chen et al., 2014), strengthen their connections with others, and deepen embeddedness in the organization, so as to generate a strong sense of belonging and increase loyalty to the profession (Halbesleben and Wheeler, 2008). Moreover, previous studies have reported that challenging work provide opportunities for personal growth and fulfillment and increase job satisfaction and well-being (Bakker and Demerouti, 2007; Podsakoff et al., 2007; Nielsen and Abildgaard, 2012), which may explain the relationship between job crafting and OC. However, there were no differences in the domain of decreasing hindering job demands among the three classes, which might be because decreasing hindering job demands may be seen more as an avoidance or distancing coping strategy than as a positive behavior in which employees try to avoid situations they perceive as stressful rather than trying to get something positive out of the situation. Evasive and distancing coping behaviors are the most ineffective (Nielsen and Abildgaard, 2012).

### 5.4 Limitations

The study has several limitations. First, we used an online questionnaire platform to recruit participants and collect data. The number of questionnaires distributed and the differences between nurses who participated and those who declined based on the information received were not clear. Second, the use of self-reported measures might lead to common method variance and social desirability bias. Third, the lack of a measure of turnover intention.

## 6 Conclusion

The study indicated three latent patterns of OC-the Low, Moderate, and High OC groups, with the “Moderate OC group” predominating in nurses. In addition, it suggested level of education, work unit and length of service were the important risk factors of “Low OC group.” The findings provided useful insights for nursing managers to better understand the profiles of OC of nurses in China, to detect the nurses who were at lower risk of OC and to be able to intervene early. Further research on facilitating meaning and job crafting should be considered. The key for managers is to help nurses recognize how their values align with the work and adjust accordingly when needed.

## Data availability statement

The raw data supporting the conclusions of this article will be made available by the authors, without undue reservation.

## Ethics statement

The studies involving humans were approved by the Ethics Committee of Xi’an Jiaotong University Health Science Center (No. 2022-1175). The studies were conducted in accordance with the local legislation and institutional requirements. The participants provided their written informed consent to participate in this study.

## Author contributions

ZL: Conceptualization, Investigation, Methodology, Software, Writing – original draft. WW: Investigation, Writing – review & editing. HZ: Investigation, Writing – review & editing. ZH: Investigation, Writing – review & editing. MH: Investigation, Writing – review & editing. JL: Investigation, Methodology, Project administration, Writing – review & editing.

## Footnotes

^1^ www.wjx.cn
